# Gender Differences in the Neurobiology of Anxiety: Focus on Adult Hippocampal Neurogenesis

**DOI:** 10.1155/2016/5026713

**Published:** 2016-01-14

**Authors:** Alessandra Aparecida Marques, Mário Cesar do Nascimento Bevilaqua, Alberto Morais Pinto da Fonseca, Antonio Egidio Nardi, Sandrine Thuret, Gisele Pereira Dias

**Affiliations:** ^1^Translational Neurobiology Unit, Laboratory of Panic and Respiration, Institute of Psychiatry, Universidade Federal do Rio de Janeiro, Avenida Venceslau Brás, 71 Fundos, Praia Vermelha, 22290-140 Rio de Janeiro, RJ, Brazil; ^2^Physics Institute, Universidade Federal do Rio de Janeiro, Avenida Athos da Silveira Ramos, Cidade Universitária, 21941-916 Rio de Janeiro, RJ, Brazil; ^3^Laboratory of Adult Neurogenesis and Mental Health, Department of Basic and Clinical Neuroscience, Institute of Psychiatry, Psychology and Neuroscience, King's College London, London SE5 9RT, UK

## Abstract

Although the literature reports a higher incidence of anxiety disorders in women, the majority of basic research has focused on male rodents, thus resulting in a lack of knowledge on the neurobiology of anxiety in females. Bridging this gap is crucial for the design of effective translational interventions in women. One of the key brain mechanisms likely to regulate anxious behavior is adult hippocampal neurogenesis (AHN). This review paper aims to discuss the evidence on the differences between male and female rodents with regard to anxiety-related behavior and physiology, with a special focus on AHN. The differences between male and female physiologies are greatly influenced by hormonal differences. Gonadal hormones and their fluctuations during the estrous cycle have often been identified as agents responsible for sexual dimorphism in behavior and AHN. During sexual maturity, hormone levels fluctuate cyclically in females more than in males, increasing the stress response and the susceptibility to anxiety. It is therefore of great importance that future research investigates anxiety and other neurophysiological aspects in the female model, so that results can be more accurately applicable to the female population.

## 1. Introduction

Anxiety and fear are adaptive emotional reactions to both innate and conditioned stimuli perceived as dangerous. They have likely been conserved throughout evolution for their adaptive value in the survival of species by warning the individual of potential dangers through the triggering of a series of neurochemical, neuroendocrine, and behavioral responses. However, when these reactions become constant and intense, with prolonged or inadequate responses to neutral stimuli or even in the absence of stressors, it may be indicative of pathological anxiety [1, 2].

Anxiety disorders cause great suffering and loss of quality of life [3, 4]. A substantial literature suggests that women may be more vulnerable than men to developing anxiety [3–6]. Anxiety disorders are diagnosed at least twice as often in women than in men, and the prevalence in women increases with age, with the gradual decline of estrogen E2 secretion from the ovaries at menopause [7, 8]. Generalized anxiety disorder (GAD), for example, occurs in approximately 5% of the population; however, the incidence doubles in postmenopausal women [9].

**Figure 1:**
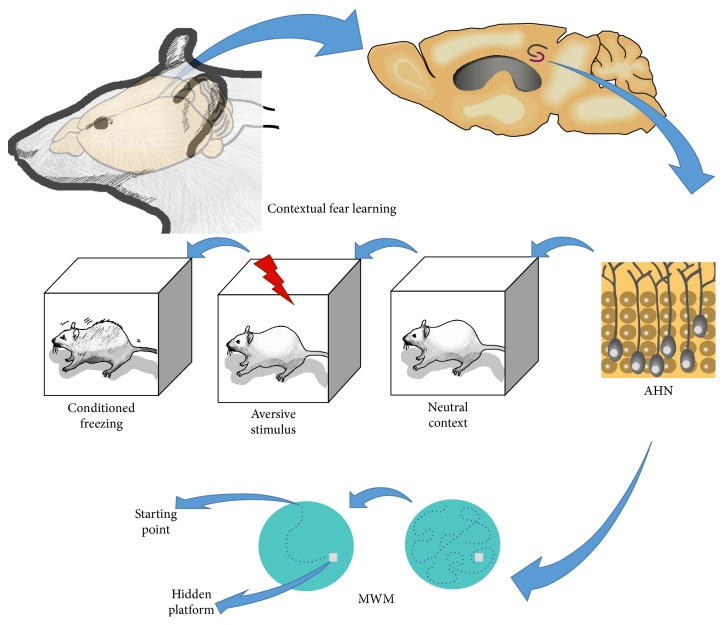
AHN is important for cognitive and emotional learning. The newly born neurons continuously generated in the postnatal hippocampus are believed to regulate cognitive and emotional tasks, as occurs in the contextual fear learning paradigm and the spatial learning assessed in the MWM. In contextual fear learning, the hippocampus is thought to be essential for the association between a previously neutral context and an aversive stimulus (in this case, a mild footshock) leading to a fear response (conditioned freezing) when the individual is reexposed to the context where the fear learning occurred. In the case of spatial learning, as assessed by the MWM, hippocampal cells are believed to play an important role in the cued spatial navigation strategies that make it possible for the rodent to more quickly find the hidden platform across the test trials. AHN = adult hippocampal neurogenesis; MWM = Morris water maze.

At the neurobiological level, the basis of anxiety can be conceived of as a disruption in the fundamental mechanism of fight and flight responses regulated by the hypothalamic-pituitary-adrenal (HPA) axis. Fear and anxiety, therefore, involve brain structures participating in the regulation of the HPA axis, such as the amygdala, the hypothalamus, the periaqueductal grey, and the hippocampus [10]. Here, we highlight this latter structure and its remarkable ability to generate newly functional neurons throughout life, a phenomenon called adult hippocampal neurogenesis (AHN). Besides their well-known functions in regulating cognitive processes, these newly generated neurons have also been implicated in the regulation of fear and anxiety [11, 12] (Figure 1).

In addition, evidence suggests that both progesterone [13] and estrogens [14, 15] play an important role in enhancing the proliferation and survival of new neurons in the hippocampus of adult females, with ovariectomized (OVX) rats displaying impaired AHN [16]. Estrogens, particularly E2, play an important role in brain development, functioning, and aging; in addition, they exert important antioxidant [17], anxiolytic, and antidepressant-like effects [9, 18–20] besides modulating the dopaminergic [21, 22], serotonergic [23], and cholinergic neurotransmitter systems [24]. The cyclical nature of the secretion of estrogen until menopause, when women experience its almost total withdrawal, supports the role of hormones in gender differences and may contribute to the greater vulnerability of women to anxiety disorders at this age. However, in animal models, both at the behavioral and at the neurogenic levels males and females may respond differently, depending on the treatment, age, or exposure to stressors used, as will be seen in this review. This leads to a lack of a clear understanding on whether males and females display different neurogenic profiles either at baseline or under different experimental conditions. Such possible differences in neurogenesis could account at least in part for the differences in anxiety observed between genders in clinical practice. Therefore, considering the importance of understanding gender differences in the context of anxiety, so that more tailored interventions may be delineated for the women population, it is essential to discuss the evidence on the possible biological differences between males and females; here, a special focus is given to AHN and anxious behavior.

## 2. Neurobiology of Anxiety: Differences between Males and Females

Overall, women and men are physiologically very similar, except for the time, pace, and schedule of the production and secretion of certain hormones. Both genders are undifferentiated until the sixth week of gestation, when the testicles develop in males and the production of androgens begins, while in females a substantial increase in follicle stimulating hormones (FSH) takes place around 12 to 20 weeks. After this period, this process of sexual differentiation is terminated, and the hormonal environment of the brain is again very similar in males and females until puberty [7].

Estrogen is a crucial hormone for the regular functioning of the brain, and its exhaustion at menopause may contribute to the higher probability of development of pathological anxiety [25]. OVX rat models have been widely used to investigate the effects of reduced estrogen levels at menopause, although the fact that it induces a drastic decline in estrogen secretion, whereas at menopause this process occurs gradually, is an important limitation of the model. The aged rodent is another useful model, but less used for this purpose. Aged female mice have very low levels of E2 and experience increased anxiety that is associated with the decline in ovarian function [9].

Hormonal differences also play a role in levels of stress markers. Females present elevated HPA axis markers at both resting and stressed states [26], as well as higher baseline plasma corticosterone (CORT) levels in comparison to males [27]. In addition, greater CORT response has been demonstrated in females than males in some anxiety tests such as the elevated plus-maze (EPM) and the defensive prod-burying test even after treatment with diazepam [28].

Additionally, CORT levels in females are influenced by the estrous cycle peaking in the proestrus phase and declining during the estrous phase, which at least partly explains why males and females respond differently to stressful situations [29] (for more details on the estrous cycle, please see Box 1).

Although clearly important, the influence of sex hormones is not the only mechanism involved in the development of sexual dimorphism. Genetic mechanisms, regardless of hormone action, may trigger the sexual differentiation of the brain and behavior [5]. The environment also appears to have an important impact on the dimorphism and differentiation of the central nervous system (CNS), thus also affecting behavior [5, 30]. In addition, sex differences in AHN (as will be later discussed) can also differently influence sexual dimorphisms.

Growing evidence also points to a role of gastrointestinal (GI) activity in leading to differential behavioral and neuroendocrine changes in males and females. Research data show that gastric inflammation induced by iodoacetamide (IAA) leads to anxiety behavior in female rats by a neuroendocrine pathway (the HPA axis), but not in male rats [27]. The reduction in circulating CORT, in the mRNA expression of glucocorticoid receptors (GR), and the increase in corticotrophin-releasing factor (CRF) mRNA in the hypothalamus of IAA-treated females suggest that GI activity has a gender impact on the HPA axis, being associated with its hyperactivity in females. This hyperactivity, in turn, is likely due to different sensitivities in the negative feedback of the HPA axis by CORT [27].

Gender differences were also found in the maternal separation model in rats, where separation during the lactation period resulted in decreased anxiety in females in comparison to males, despite the decreased expression of gamma-aminobutyric acid- (GABA-) A receptors in both sexes [31].

Significant gender differences were also reported in the prelimbic (PL) cortex activity during fear extinction and extinction recall [32]. Males presented PL activity decreased in the safe context, while females displayed increased PL activity in the same context; however, both showed increased infralimbic (IL) cortex activity before extinction recall compared to before extinction. These results suggest that female rats show increased expression of learned fear involving the persistent activation of PL.

According to the authors, this result may be related to possible disruptions in hippocampal connectivity to the PL, considering the role of the hippocampus in mediating contextual processing. In particular, the input of the ventral hippocampus (VH) to PL appears to be linked to the regulation of the extinction process [33]. In this sense, it has been shown that the temporary inactivation of the VH increases PL activity and expression of learned fear after extinction [34]. The authors highlight that females could therefore display changes in hippocampal-mediated inhibition of mPFC function, in agreement with findings in female patients with posttraumatic stress disorder (PTSD) [32, 35]. Furthermore, anxiety has been linked with impaired hippocampal neurogenesis [12], raising the possibility that altered AHN could also participate in the regulation of the fear circuitry. Whether this could be related to differential rates of AHN between males and females is a question that will be further discussed next.

### 2.1. Adult Hippocampal Neurogenesis and Anxiety

Adult neurogenesis, a phenomenon first described by Altman during the 60s [36], refers to a mechanism of continuous formation of newly functional neurons throughout life, a process that takes place only in specific regions of the adult brain [37].

In this regard, although also identified in structures such as the hypothalamus and the amygdala [38], neurogenic niches in the adult brain are mainly considered to reside in the dentate gyrus (DG) of the hippocampus and the subventricular zone (SVZ) adjacent to the lateral ventricles [39, 40].

Due to its influence on mental health-related behaviors, including anxiety, we will focus this review on hippocampal neurogenesis.

The hippocampus is a region extremely sensitive to stress, and neurogenesis has been proposed to be linked to the development of pathological anxiety [41]. Growing evidence supports this idea, as chronic stress has been shown to reduce hippocampal neurogenesis, besides changing the activity of the HPA axis, thereby undermining the ability of the hippocampus to modulate the brain areas involved in stress and anxiety responses [42, 43].

AHN is a dynamic and highly regulated process, comprised of the stages of proliferation, differentiation, migration, integration, and survival [37, 40]. The subgranular zone (SGZ) of the DG is known to contain a large number of neural progenitor cells (NPCs) that retain the ability to divide resulting in cells that have the potential to become mature granule cells [44]. Proliferation is an expansion of the pool of NPCs, followed by a selection process where about half of these new cells will undergo apoptosis [45], while the surviving neuroblasts migrate into the granular zone of the DG [46]. Mice studies have shown that around four weeks these new cells already begin to express neuronal markers and are functionally incorporated into the preexisting circuit [47].

This mechanism of generation of new neurons can be affected by external stimuli, such as learning and memory [48], exercise [49], diet [50], environment [51], stress [52], and aging [53], as well as pharmacological agents [54] and internal stimuli. Among the internal stimuli known to upregulate the AHN process, we can highlight the microenvironmental factors, such as trophic factors [55], growth factors [56], increased vasculature [48], chemical and electrical changes such as excitatory stimuli [57, 58], and gonadal hormones (estradiol in females and testosterone in males) [59]. Moreover, there are also microenvironmental factors that negatively regulate this process, such as immune responses [60], glucocorticoids [42], and physiological changes associated with aging [61].

In addition, recent studies have attributed to AHN a regulatory role in various cognitive processes such as memory [42, 62], learning [61, 63, 64], besides a significant influence on affective disorders [65], anxiety [12], and emotional behavior [66]. Evidence for the role of AHN in the regulation of learning and memory is based on electrophysiological findings showing that new neurons in the hippocampus exhibit enhanced long-term potentiation (LTP), an important cellular mechanism underlying learning processes and memory [67]. In accordance with this, studies have shown that ablation of neurogenesis using genetic manipulation or irradiation techniques results in behavioral changes, such as cognitive deficits [68], including impaired performance acquired in the water maze by runners mice [69].

Despite the classic role attributed to the hippocampus and AHN on cognition, several researchers have suggested a link between neurogenesis and anxiety-related behaviors. One of the most convincing studies demonstrating this association was published by Revest et al. [12]. In this study, the authors used an inducible transgenic strategy that enabled the specific ablation of newborn neurons in the adult DG to demonstrate that deficits in hippocampal neurogenesis lead to an increase in anxious behavior. Furthermore, Dias and colleagues have found a decreased number of immature neurons in the DG of a rodent model for the study of generalized anxiety [70]. In addition, it has been suggested that the birth of new neurons in the hippocampus may be involved in the ability of the DG to distinguish contexts, and deficits in this ability could be an important factor in the etiology of anxiety disorders [11, 71]. The promotion of AHN has therefore been discussed as a potential target for the treatment of anxiety disorders [65, 71, 72].

### 2.2. Adult Hippocampal Neurogenesis: Differences between Males and Females

The differences between sexes with regard to AHN are derived largely from the singular physiology of females that allows pregnancy, parturition, and lactation [73]. This particular physiology makes females undergo profound hormonal changes throughout life. In addition, hormonal fluctuations experienced by females during biological processes such as the estrous cycle, pregnancy, and maternity are associated with a number of changes in the brain and behavior [74].

Gonadal hormones, besides exerting a powerful anxiolytic role, are also known by their effects in directly affecting hippocampal neurogenesis in females, via regulation of the proliferation and survival of new neurons [59]. This effect is possible due to the presence of estrogen receptors, ER*β* and ER*α* in the DG [59, 75]. Evidence has shown that both ER*α* and ER*β* receptors are involved in the improvement of AHN in rats [26, 76]. However, ER*β* agonists result in a greater neurogenic response, suggesting that ER*β* may be more strongly associated with AHN than ER*α* [75]. Conversely, the ER antagonist ICI 182,780 could partially block the rise in estradiol-induced cell proliferation in the DG of rats [38]. Luteinizing hormone (LH), known for its important role during pregnancy and sexual behavior, also exerts influence on neurogenesis in females. Studies have shown that exposure of females to male pheromone or subcutaneous administration of a high dose of LH induced increased proliferation of new neurons both in the SVZ and in the DG [77]. Induced sexual experience was also reported to increase neurogenesis and reduce anxiety in females [78].

Although other female gonadal hormones like progesterone [73, 79] and LH [77] can also lead to improvements in AHN, estrogens are the most widely studied class of gonadal hormones, especially estradiol, the most potent form of estrogen [75]. Several articles have shown significant increases in the proliferation and survival of newly born neurons in the hippocampus after treatment with estradiol [13, 59, 73, 76, 80, 81], but not with other estrogens, such as estrone [14, 75]. In addition, during the proestrus phase of the estrous cycle, where estradiol levels are higher, rats exhibit about 50% higher proliferation than rats during diestrus and estrus stages when estradiol is low [82]. In OVX rats, proliferation of new neurons is significantly reduced, while estrogen replacement reverses the effect of ovariectomy [38, 83]. Additionally, another study has shown increased proliferation of new DG cells in OVX rats after estradiol injection or addition of soy extract in drinking water [84]. Furthermore, estrogen can also mediate AHN via growth factors and/or neurotransmitter systems.

Evidence suggests that estrogen regulates the expression of BDNF which in turn promotes the survival of newborn neurons in the hippocampus [85]. Moreover, it is known that estrogen plays an important role in modulating the levels of serotonin (5-hydroxytryptamine; 5-HT) synthesis via the regulation of tryptophan hydroxylase [74]. It has been shown that an increase in 5-HT in the DG increases cell proliferation while a reduction of 5-HT reduces proliferative activity [38]. In addition, serotonin antagonists can block the effect of estradiol to enhance cell proliferation [59, 86]. Also, it is well established that animal models of anxiety have exaggerated HPA axis responses to stress. In this context, it has been shown that E2 administration to rats with low endogenous levels of this hormone can alter this response, but its effects are previous experience- and regimen-dependent [9].

Estrogen levels have, therefore, been positively correlated with cell proliferation and negatively correlated with cell death [83]. During pregnancy and* postpartum* period, for example, estrogen is related with an increase of neuroplasticity [83]. During the proestrus phase, which is characterized by high levels of circulating estrogen, females exhibit greater cell proliferation in the DG than males, or females during the other phases of the estrous cycle [87, 88]. Moreover, several studies have shown that females and males respond differently to gonadal hormones administration in the context of hippocampal neurogenesis (see Table 1).

Treatment with estradiol or estradiol benzoate produced different behavioral effects in both genders [80, 89]. With regard to neurogenesis, females after estradiol benzoate treatment exhibited an increase in cell proliferation, and a decrease in both overall cell death and neuron survival in the DG, but males are minimally affected [80]. On the other hand, males injected with estradiol for 30 days presented no change in neurogenesis; however, they showed significant increased AHN via cell survival after treatment with both testosterone and dihydrotestosterone (DHT), one of the main metabolites of testosterone [90]. In addition, rats treated with the organochlorine insecticide methoxychlor (MXC), a synthetic compound known for their xenoestrogens properties able to cause disruption of the endocrine system, exhibited higher density of surviving cells in males than in females [91].

Sex differences in regard to AHN with respect to the stress response have also been widely reported. Exposure to stress increases levels of glucocorticoids, and when occurring during the prenatal period, this increase can cause substantial changes in neuroplasticity, reducing the capacity for cell proliferation in adults [92]. Accordingly, analysis of the brain tissue of adult rats whose dams were subjected to restraint stress 3 times per day during the last 10 days of pregnancy showed decreased survival of new neurons in the DG and increases of hippocampal BDNF levels in males, but not in females [93]. Besides, the type of stress and the duration (whether acute or chronic) are also an important factor in influencing AHN. Both acute stress caused by foot-shock [94] as caused by acute exposure to a predator odor have been reported to be associated with reduced cell proliferation in the male, but not in the female hippocampus [95]. Conversely, individually housed female rats which underwent chronic stress (daily foot-shocks for 3 weeks) showed increased BrdU labeling in comparison to males [82].

The age of animals is another relevant variable for the understanding of gender differences in the context of AHN. Postnatal neurogenesis during puberty occurs in young animals (between postnatal days (PND) 21 and 28; PND21–28) who have not yet reached sexual maturity [96]. Investigations of neurogenesis at this stage of development are important for the understanding of the mechanisms underlying neurogenesis and evaluation of their possible changes and particularities throughout life. Hodes et al. compared the effects of chronic fluoxetine treatment in adult and peripubescent rats of both sexes and found an increase in cell proliferation in adult males but not in adolescent males or females, as well as reduced cell survival in females but not in males [97]. Investigations about the effects of maternal deprivation in rats in early life reported increased proliferation in the DG in males and decreased in females at PND21 [98]. However, in an experiment in which male mice were subjected to early life stress (maternal separation) it was found that rats tested during peripubescence (PND21) exhibited increased neurogenesis, whereas when tested during adulthood (2 months old) no differences were found, and at middle-age (15 months old) AHN was decreased [99]. Moreover, using a different type of early life stressor (limited nesting and bedding material from PND2–9), Naninck and colleagues showed that both sexes exhibited significantly increased proliferation at PND9, but in adulthood males presented reduced long-term survival of newly generated cells, while females were not affected [100].

The effects of early life stress thus appear to be sex- and age-dependent, with different responses depending on the age at which the hippocampus is under analysis. Increased neurogenesis in young rodents which were subject to early stress may be explained as a compensatory mechanism enabling the survival of the organism under adverse conditions. However, this early improvement is not always observed in females. In addition, it may be interesting to note that the decreased neurogenesis reported in some studies in females using early life stress occurs during a critical period of brain development, which could increase the vulnerability of females to the development of psychiatric disorders such as depression [101], a condition often found to be comorbid with anxiety. A reduction in hippocampal volume in women who experienced early childhood trauma has been reported, suggesting that stress early in life can alter the structure and function of the hippocampus in humans also [102]. Whether this reduced hippocampal volume is also associated with reductions in AHN is a question yet to be investigated.

## 3. Potential Interventions for the Enhancement of AHN and Alleviation of Anxiety

Several external factors can induce physiological changes in the organism, thus exerting influence over AHN rates [38, 108, 109]. Based on this knowledge, different interventions have been proposed as possible enhancers of this mechanism [9, 38, 45, 110]. Here, we highlight some of them as possible ways to help overcome anxious symptoms, although it is clear that further studies are needed in order not only to clarify the participation of AHN as a pivotal mechanism underlying the higher anxiety observed in the women population but also to ascertain the effectiveness of these interventions among females.

Exercise is cited as one of the most powerful stimulants of neurogenesis [111–114]. It is believed that this effect is due to the increased oxygenation, metabolism, and blood flow favored by exercise, which could result in an increased nutrient delivery, providing increased synthesis and release of growth factors, such as brain-derived neurotrophic factor (BDNF), and neurotransmitters [115–117]. Furthermore, physical exercise has been reported to reduce anxiety. Corroborating this idea, studies have shown that voluntary wheel running produced anxiolytic effects in rats and mice [118–120], while the cessation of voluntary wheel running increased anxiety and impacted AHN negatively [121]. This strongly indicates that the improvement of neurogenesis afforded by exercise is associated with reduced anxious symptoms [121], although excessive physical exercise, at least in male mice, has been shown to improve neurogenesis but also anxiety-like behavior [122]. Furthermore, depending on the context, voluntary exercise can accentuate anxiety in females, as is the case with treatment with androgenic anabolic steroids [123]. Further studies are therefore needed in order to unravel the optimal conditions where females can benefit from physical exercise as an intervention to reduce anxiety.

Another factor with great positive impact over AHN is diet. Dietary interventions can comprise nutrient content, quantity, frequency, and texture [50, 124, 125]. Caloric restriction (CR), for instance, has been cited as an effective intervention in the expansion of neurogenesis [50]. Studies showed that CR rats displayed an increase in AHN rates when compared to animals fed* ad libitum* [126]. In addition, CR is also aimed to improve some cognitive processes generally impaired in anxious patients, such as fear extinction learning and retention [127]. As shown by Riddle and colleagues, after CR treatment for 7 days, significant effects on the enhancement of fear extinction and retention were found only in females but not in male mice [127].

Also pointing for anxiolytic properties of CR, a study showed that male rats under a 25% CR regimen entered more in the open arms of the EPM and spent more time in the center of the arena in the open-field test (OFT), indicating reduced anxiety [128].

With respect to the frequency of food intake, studies in rodents subjected to intermittent fasting (IF), where animals are fed on alternate days, also showed improvement in brain plasticity by increasing the survival of new neurons generated [129] as well as improvement in the ability to integrate and consolidate information compared to mice fed* ad libitum* [130]. However, the literature still lacks data on mechanisms underlying the effects of IF specifically in females and its possible role in anxiety disorders. Further studies are therefore required for a deeper understanding of this intervention, especially in the female population.

With regard to the quality of nutrients, some of the beneficial compounds more thoroughly investigated as brain plasticity enhancers are the polyphenols and the n-3 polyunsaturated acids (PUFA). Polyphenols (such as curcumin and resveratrol) are bioactive compounds found in a number of plants and spices present in the human diet, such as turmeric (in the case of curcumin) and red berries, the skin of red grapes, red wine, and nuts (in the case of resveratrol). These compounds are known for their neuroprotective and antioxidants actions [131] in addition to their anxiolytic and antidepressant properties [110, 132]. The improvement of AHN as a result of polyphenolic treatment has been shown both* in vitro* and* in vivo*, with curcumin-treated mice exhibiting increased cell differentiation [133].

OVX rat models have been used to investigate the protective role of grape powder on anxiety in estrogen-deficient females. OVX rats treated with grape powder for 3 weeks showed decreased anxiety [134]. In addition, a study in OVX mice suggested that resveratrol could act as an ER agonist mimicking the effects of estrogen [135], which potentially could be used as a safe alternative to protect the brain against the effects of estrogen deficits that occur in menopause.

As well as treatment with polyphenols, the consumption of foods rich in PUFAs has also been shown to exert positive effects on neurogenesis. A study by Venna et al. found an increase in cell proliferation in the DG after 5 and 6 weeks of PUFA supplementation [131], highlighting the important role of diet in brain plasticity, and AHN in particular.

A number of studies have suggested that an enriched environment (EE) is a promising intervention for the improvement of AHN [136–138]. EE consists of a larger habitat where animals are housed in groups so that social interaction is facilitated. This environment is also characterized by the presence of stimulating toys, tunnels, and platforms. These stimuli are changed regularly in order to promote curiosity and exploration, as well as to provide sensorial experience, and motor and cognitive stimulation to the animals. With regard to AHN, a study by Leal-Galicia et al. showed increased proliferation, survival, and differentiation of new neurons in the DG of rats submitted to EE, as well as reduced anxiety in several behavioral tests accompanied by higher rates of BrdU positive-cells in the hippocampus of animals with a reduced anxiety response [139]. In accordance with these findings, several studies have found an improvement in neuroplasticity accompanied by reduced anxiety in rodents subjected to EE paradigm [140–142]. However, findings suggest that the duration of exposure to an EE seems to influence anxiety-like behaviors. For example, reduced anxiety in the EPM was observed in mice exposed to an EE for 3 weeks, but not in the group exposed for 24 hours, 1 week or in animals subjected to more prolonged periods of exposure, such as five weeks [143]. However, not all these interventional studies were undertaken with female rodents. This highlights the need for future investigations into the effects of these potential interventions on reducing anxiety-like behavior in this population both in AHN-dependent and independent manners.

Another possible category of intervention could be hormonal therapy. E2-based therapies have been used for many years to treat physiological symptoms associated with menopause such as hot flushes, sweating, genital dryness, and mental symptoms, such as cognitive deficits and increased anxiety [20]. Several studies in menopausal women have shown that E2 replacement therapy attenuates the loss of cognitive performance associated with the end of the reproductive cycle [9]. Women who received E2 after menopause demonstrate verbal improvement, and improvement in short and long-term memory, as well as logical reasoning, compared to controls [144, 145]. In animal models, it has been shown that cognitive deficits related to reduction of circulating E2 levels after menopause coincided with the reduction of cell proliferation in the hippocampus [83]. On the other hand, increasing levels of estradiol have been suggested to improve neurogenesis and cognitive aspects. As shown by a study by Frye et al., E2 administration to mice reverses the cognitive deficits caused by aging, significantly improving spatial learning performance and reference memory in the water maze task [146].

However, the limitations of hormone replacement therapies due to their proliferative effects on breast and uterus curbed the enthusiasm of the use of E2 as treatments for anxiety disorders [147]. Moreover, not all individuals respond favorably to E2. Some women with anxiety disorders report less anxiety when E2 levels are low and/or relatively stable, which suggests that some individuals may be more sensitive than others to E2 [9, 148]. For these individuals, other strategies such as diet based on phytoestrogens, compounds present, for example, in soy extract with weak estrogenic or antiestrogenic activity, might be useful considering their neuroprotective effect, and their ability to increase the production of new cells in the hippocampus [84]. Another phytoestrogen, *α*-zearalanol (*α*-ZAL), has been used as a safe alternative for estrogen, due to reduced side effects on the uterus and breast. Studies showed that both *α*-ZAL and 17*β*-E2 improved neurogenesis and learning and memory deficits [149].

Data from a recent study, though, showed that treatment with 10b, 17b-dihydroxyestra-1,4-dien-3-one (DHED) was effective in reducing symptoms associated with estrogen deficits in the brain and in promoting neuroprotective effects in rats [150]. These are promising findings, considering that DHED is a small-molecule bioprecursor prodrug which is converted to 17b-estradiol in the brain but remains inactive in the rest of the body. This, therefore, prevents the adverse side effects normally found in the periphery and for which reason estrogen replacement therapies cannot be used safely.

Besides, as previously discussed, it could be hypothesized that cases unresponsive to E2 could still benefit from other practices such as those related to diet, exercise, and a stimulating environment. Novel studies are, however, still needed to assess their efficacy in terms of onset, duration, and synergistic effects with other interventions and conditions in humans, with a special focus here on the female population.

## 4. Discussion

Animal models can help to elucidate some aspects of neuropsychiatric disorders, but their establishment implies some important principles. Behavioral models can be appropriate for one sex and inappropriate for another, and generalizations on the findings in one sex to another seem to be a biased process at best. Therefore, caution is needed when interpreting data, as it may be possible that certain behavioral paradigms and interventions are not interchangeable in males and females [5]. In addition to behavioral differences, there are outstanding physiological differences between genders, as indicated throughout the paper. The reproductive process results in important functional changes in the female brain, mainly due to gonadal hormones. AHN, for example, is regulated in females and males by both gonadal and adrenal hormones, but are sex- and experience-dependent [73]. This shows that it cannot be affirmed that males and females have each a certain established neurogenic profile, as this changes in accordance with a number of variables. Moreover, results of behavioral or physiological analysis in females depend on the stage of the estrous cycle, that is, the hormone levels that are circulating at that moment [83, 89]. Finally, there is a very small number of studies in the literature assessing gender differences in behavioral tests of anxiety. Therefore, the hypothesis raised in this review will only be fully answered by future studies on the possible mechanisms underlying the gender gap with regard to stress or threat responses, as well as using AHN markers as one of their neurobiological readouts. These will be an invaluable source to help us better understand the differential vulnerability for mood and anxiety disorders between men and women [7].

Clinical studies showed that due to the sudden drop of estradiol levels that occurs during pregnancy, postpartum women have a higher reactivity of the HPA axis to stressors [27, 151]. Furthermore, estradiol is pointed to modulate neurotransmission, synaptic plasticity, and neurogenesis [75, 152], besides cognitive functions and emotional responses through the hippocampus, a structure known as one of the key regions of the so-called emotional brain due to its role in modulating anxiety states [12, 153]. There is growing evidence showing that deficits in neurogenesis are related to increased anxiety-like behavior [12, 154–157]; on that account, investigating the mechanisms of action and effects of E2 on the modulation of AHN and anxiety disorders has become a goal of potentially great importance and clinical impact. In recent years, a significant increase in life expectancy of women has been observed; however, the age of onset of menopause has remained relatively constant, resulting in a larger portion of life, about one-third, where women live with low endogenous levels of E2. It is therefore likely that more women make use of therapies based on E2 to relieve symptoms of menopause, necessitating an intensification of research into the possible benefits and risks of hormone replacement therapy [20, 32, 158].

Functional neuroimaging techniques also appear to be a promising tool to reveal the neural mechanisms underlying anxiety disorders, leading to the development of more effective therapeutic options, as they can help us understand how new pharmacological treatment options may work and predict if the patient is likely to respond to a particular intervention or not [159]. Despite the extensive amount of research in animals, little is known about the mechanisms of neurogenesis in the adult human brain, which is limited by* postmortem* histological studies [160, 161]. Thus, the future development of more sensitive and specific techniques of molecular neuroimaging for the investigation of human AHN is of great importance, as they hold unprecedented potential for the design of more effective treatment, with less side effects and improved life expectancy and quality of life.

## 5. Conclusion

Consistent evidence in the literature points to important differences between males and females with regard to anxious behavior and a number of biological mechanisms, including AHN, with different interventions bringing both sex- and age-dependent differential regulation of the ability of the hippocampus to generate newly functional neurons throughout life. As a whole, studies with animal models support the overall idea that increased levels of AHN are associated with decreased levels of cognitive deficits and anxiety. Therefore, interventions that are able to promote AHN are hypothesized as potentially effective to improve anxiety-like behavior, although further testing in female rodents and in the human population at different ages is still needed.

Of special note, disrupted levels of estrogen at menopause may contribute to the development of pathological anxiety. In addition, with increasing life expectancy, it is likely that more women will make use of estrogen-based therapies to relieve symptoms of menopause, making it necessary that research in females be undertaken into the possible benefits and risks of hormone replacement therapy, as well as on interventions that may enhance AHN and alleviate symptoms of anxiety.

## Figures and Tables

**Box 1:**
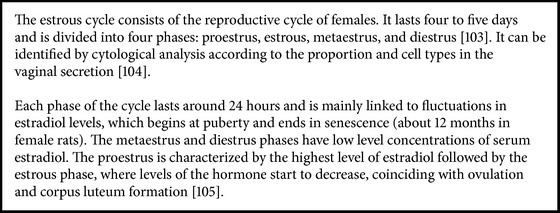
**Box 1: **Estrous cycle.

**Table 1:** Differences between males and females with regard to AHN.

Model	Intervention/behavioral paradigm	Differences in AHN between males and females	Differences in anxious behavior between males and females	Differences in other types of behavior between males and females	Reference
PND70–90 Sprague-Dawley rats	Eyeblink conditioning	Twice more new cells (mainly neuroblasts) survived in the female than in the male hippocampus	—	Females learned to time the conditioned response faster than males	[63]

380 g (male) and 240 g (female) Wistar rats	Exposure to stressors	↑ proliferation in the DG of females compared to males; ↑ DCX in the DG of males compared to females	—	Females showed ↑ basal and stress-induced HPA axis activity compared to males	[87]

PND80–90 Sprague-Dawley rats	Treatment with E2 or sesame oil	↑ proliferation, ↓ cell death, and ↓ survival in the DG of females; males were affected minimally	—	Female rats froze less than males after contextual fear conditioning	[106]

PND80–90 Sprague-Dawley rats	Treatment with E2 benzoate	↓ survival, ↑ proliferation, ↓ cell death in the DG of females, and no effect in males	—	—	[80]

3-month-old Wistar rats	Chronic stress	↓ BrdU labelling in males, but ↑ in females	—	—	[82]

250–300 g Sprague-Dawley rats	Acute stress (exposure to a predator odor)	↓ proliferation, ↓ cell death in males but not in females	—	—	[95]

PND58–62 Sprague-Dawley rats	Spatial task	↑ BrdU-labeled cells in males, ↑ cell activation in females but not in males	—	Males performed better in the spatial but not cue task than females	[107]

2-3-month-old Sprague-Dawley rats	Acute stress	↓ proliferation in male hippocampus but not in female	—	Exposure to stress significantly ↓ learning ability in females but ↑ in males; males expressed more helplessness behavior than females	[94]

2-3-month-old Swiss CD1 mice	Treatment with MXC	↑ survival in males compared to females		Male mice exhibited ↓ contextual conditioned freezing compared to females	[91]

3-month-old Sprague-Dawley rats	PRS	↓ number of new neurons in the DG and ↑ BDNF levels in males but not in females	Males showed ↑ anxiety, while females displayed ↓ anxiety in the EPM	—	[93]

PND63–65; PND24–26 (peripubescent) Sprague-Dawley rats	Fluoxetine treatment	↑ cell proliferation in males but not in females ↓ cell survival in females but not in males	—	—	[97]

C57Bl/6J mice	ES (limited nesting/bedding material)	↓ cell survival only in males	—	Males showed impaired cognitive performance in the ORT, OLT, and MWM, compared to females	[100]

AHN = adult hippocampal neurogenesis; BDNF = brain-derived neurotrophic factor; BrdU = bromodeoxyuridine; DG = dentate gyrus; DHT = dihydrotestosterone; DCX = doublecortin; EPM = elevated plus maze; ES = early life stress; E2 = estradiol; MXC = methoxychlor (organochlorine insecticide); PND = postnatal day; PRS = prenatal restraint stress; SVZ = subventricular zone; T = testosterone; ES = early life stress; ORT = object recognition task; OLT = object location task; MWM = Morris water maze.
